# Advancements in the physiopathological study of acupuncture treatment for insomnia: A review

**DOI:** 10.1097/MD.0000000000038476

**Published:** 2024-06-28

**Authors:** Haishen Zhao, Chengjun Liu, Hong Ye, Kejun Shi, Yijie Yao, Manya Wang

**Affiliations:** a Rehabilitation Department of Luchaogang Community Health Service Center in Pudong New Area, Shanghai, China.

**Keywords:** acupuncture, insomnia, mechanism, physiopathological

## Abstract

Insomnia is a common sleep disorder that significantly impacts an individual’s psychological and physical health, characterized by persistent difficulties in falling asleep, decreased sleep quality, and impaired daytime functioning. Traditional treatment approaches primarily rely on pharmacotherapy and behavioral therapy, yet not all patients benefit from these methods, and they often come with certain side effects. Thus, identifying safe and effective alternative or adjunctive treatments is of paramount importance. The purpose of this paper is to review the research progress on acupuncture in the treatment of insomnia and to explore the mechanisms by which acupuncture may treat insomnia through regulating neurotransmitters and nervous system, anti-inflammatory actions, and improving neuroplasticity, providing evidence to support the broad application of acupuncture in clinical practice.

## 1. Introduction

Insomnia is a sleep disorder characterized by difficulties in falling asleep or maintaining sleep. Some individuals experience both issues, while others may wake up early in the morning and find themselves unable to fall back asleep. Significantly, these nighttime sleeping challenges are coupled with severe daytime issues that impair optimal functioning, including daytime fatigue, mood disturbances such as feeling down or irritable, and problems with concentration or focus.^[1]^

Under the International Classification of Diseases, Eleventh Revision, insomnia is categorized within a broader spectrum of sleep-wake disorders, identified as a series of distinct disorders.^[2]^ Specifically, insomnia is classified into 2 main types: Chronic Insomnia Disorder and Short-term Insomnia Disorder.^[3]^ The previously used terms “primary” and “secondary” insomnia, along with different subsets of chronic insomnia such as “psychophysiological insomnia,” have been replaced by the more encompassing term “Insomnia Disorder.”

Chronic insomnia is characterized by frequent and persistent difficulties in sleeping despite adequate opportunities and conditions for sleep, leading to general dissatisfaction with sleep and some form of daytime impairment. Daytime symptoms often include fatigue, mood disturbances such as depression or irritability, physical discomfort, and cognitive impairments. Sleep disturbances and associated daytime symptoms occur several times a week and last for at least 3 months. Some individuals with chronic insomnia may experience a more episodic course, with periods of sleep/wake difficulties recurring over several weeks or persisting for years. Short-term insomnia is characterized by difficulty initiating or maintaining sleep for less than 3 months, despite adequate opportunities and conditions for sleep. This condition leads to general dissatisfaction with sleep and some form of daytime impairment. Daytime symptoms typically include fatigue, mood disturbances such as depression or irritability, physical discomfort, and cognitive impairments. Individuals who report sleep-related symptoms without significant daytime impairment are not considered to have insomnia. Insomnia should be diagnosed as either chronic or short-term only if it is the primary focus of clinical concern, not attributable to another sleep-wake disorder, mental disorder, medical condition, or substance/medication use.^[4]^

The etiology of insomnia is multifactorial, involving complex interactions among biological, psychological, and environmental factors. These may include genetic predispositions, neurobiological imbalances, psychological stress, and lifestyle choices. Despite the high prevalence of insomnia, it often remains underdiagnosed and undertreated due to the limitations of current treatment methods and patients’ reluctance to seek treatment.^[5]^

Traditional treatment approaches for insomnia primarily focus on pharmacotherapy and cognitive behavioral therapy for insomnia (CBT-I). In the acute treatment phase, a synergistic effect can be observed between CBT-I and pharmacotherapy^[6]^; however, CBT-I alone has been shown to be superior to the combination of CBT-I and pharmacotherapy following acute treatment. While these treatments are effective for some, they are not suitable for all patients. Pharmacotherapy, though commonly prescribed, can lead to dependence and adverse side effects. On the other hand, despite published evidence supporting the effectiveness of CBT-I in managing insomnia and its recommendation as the first-line treatment for chronic insomnia patients, it may not be readily accessible to all due to a shortage of trained therapists or treatment costs.^[7]^

## 2. Epidemiology

Insomnia, a prevalent sleep disorder, affects approximately 10% of adults with long-term consequences, while an additional 20% experience symptoms of insomnia occasionally. As a chronic condition, the persistence of insomnia can reach up to 40% over a 5-year period. Certain demographics, such as women, the elderly, and those facing socioeconomic challenges, are more susceptible to insomnia.^[8]^

A literature^[9]^ search conducted in databases revealed studies on the prevalence of insomnia based on gender differences. The results indicate that the overall prevalence rate of insomnia is 22.0%, with women experiencing a significantly higher rate of insomnia than men, with a ratio difference of 1.58. Subgroup analyses further demonstrate that studies using case-control designs and consecutive sampling methods show a larger gender difference. Moreover, meta-regression analysis indicates that a higher proportion of women in studies and higher study quality are significantly associated with greater gender differences.

## 3. Pathogenesis

### 3.1. Neurotransmitter imbalance

Neurotransmitter imbalance refers to changes in the concentration or activity of neurotransmitters in the brain, which disrupt the normal balance of neural signal transmission.^[10]^ Neurotransmitters, key chemical substances used for communication between neurons in the brain, play a crucial role in regulating emotions, cognitive functions, sleep cycles, and attention among many psychological and physiological activities. The concentration of neurotransmitters such as gamma-aminobutyric acid (GABA), cortisol, dopamine, and norepinephrine, if too high or too low, or if the sensitivity of their receptors changes, can cause a range of psychological and physiological abnormal states, including insomnia.^[11]^

GABA is considered one of the most important inhibitory neurotransmitters in the brain, playing a vital role in regulating neural excitability, maintaining emotional stability, muscle tension regulation, and sleep cycle management. By lowering neuronal activity, GABA helps promote body relaxation and alleviate tension.^[12]^ This mechanism primarily involves the opening of chloride ion channels promoted by GABA receptors, leading to the influx of chloride ions, causing the cell membrane potential to shift in a negative direction, thereby inhibiting neuronal activity and reducing brain excitability. In sleep regulation, GABA’s inhibitory action lowers the activity of the cerebral cortex, thereby promoting relaxation and sleep, crucial for maintaining normal sleep patterns.^[13]^ For individuals with insomnia, insufficient inhibitory function of GABA may lead to an inability to effectively reduce brain excitability, thus affecting the onset and maintenance of sleep.^[14]^ Additionally, a reduction in GABA activity is closely associated with the generation of deep sleep phases, further reducing deep sleep, thereby affecting sleep quality and recovery functions.^[15]^ Research^[16]^ had shown that compared to individuals with normal sleep patterns, patients with insomnia generally have lower levels of GABA in their brains. This finding reveals a potential positive correlation between GABA levels and the severity of insomnia symptoms, i.e., a reduction in GABA content seems to be directly related to the exacerbation of symptoms, further emphasizing GABA’s key role in the occurrence and development of insomnia. However, further research into this issue reveals complexity. In particular, another study proposed a mechanism contrary to reduced GABA levels, highlighting the hypothesis that a decrease in the number of GABA receptors serves as a primary cause of insomnia. In a study by Zhang,^[17]^ compared to the normal sleep control group, the mRNA expression levels of the GABAA receptor α1 and α2 subunits in the insomnia patient group were significantly reduced, although there were no statistically significant differences in serum GABA levels between the 2 groups. The study did not find a significant correlation between GABA levels and the mRNA expression of GABAA receptor α1 and α2 subunits in the insomnia patient group. Moreover, although the Pittsburgh Sleep Quality Index (PSQI) did not show a significant correlation with the mRNA levels of these 2 subunits, specific components of sleep quality and sleep duration were negatively correlated with the mRNA level of the GABAA receptor α1 subunit, while daytime function was inversely related to the mRNA level of the GABAA receptor α2 subunit. These results suggest that the inhibitory function of serum GABA in insomnia patients may be impaired, and the decrease in mRNA expression levels of the GABAA receptor α1 and α2 subunits may serve as biomarkers for insomnia.

Cortisol is a hormone related to stress responses, following distinct circadian rhythm changes. Although research results on the connection between chronic insomnia and cortisol levels vary, chronic insomnia has been considered associated with an increased risk of mental health issues.^[18]^ Research^[19]^ has observed a significant positive correlation between the Insomnia Severity Index and morning cortisol levels, indicating that individuals with higher insomnia severity exhibit higher levels of cortisol in the morning. This finding is consistent with the hypothesis that chronic insomnia is related to a high state of arousal, showing an activated stress response system in individuals with severe insomnia through elevated cortisol levels. In studying the hyperarousal state of insomnia, Roehrs and Roth^[20]^ found that cortisol levels – reflected in daytime and pre-sleep measurements of urine and saliva cortisol – were closely related to the severity of insomnia. Insomnia patients, compared to the control group, showed higher pre-sleep salivary cortisol levels, indicating a pre-sleep hyperarousal state; during the day, especially those patients who demonstrated a higher state of hyperarousal through the Multiple Sleep Latency Test, also had higher urinary cortisol levels.

5-Hydroxytryptamine (5-HT), also known as serotonin, is a key neurotransmitter found in the brain that plays a crucial role in regulating various physiological processes such as sleep, mood, appetite, and pain perception.^[21]^ The connection between 5-HT and insomnia is reflected in several key aspects: Firstly, 5-HT plays a central role in regulating the body’s circadian rhythm and sleep cycles. During the day, levels of 5-HT are higher, helping to maintain wakefulness; at night, its levels decrease, promoting sleep.^[22]^ Additionally, 5-HT is involved in regulating the transition between Rapid Eye Movement (REM) sleep and Non-Rapid Eye Movement (NREM) sleep, both of which are essential for recovery and emotional stability.^[23]^

### 3.2. Central nervous system abnormalities

Under pathological conditions, the imbalance of the autonomic nervous system (ANS) function is not only a key factor in the onset of insomnia but also increasingly recognized for its role in disease progression.^[24]^ Specifically, excessive activation of the sympathetic nervous system and reduced activity of the parasympathetic nervous system cause a range of physiological and psychological responses, including heightened heart rate and blood pressure, increased perception of stress, and exacerbated pre-sleep anxiety, all of which significantly interfere with the process of falling asleep and sleep quality.^[25]^

Insomnia patients often exhibit excessive sympathetic nervous system activation, maintaining the body in a constant “fight or flight” state, thereby increasing heart rate and blood pressure, as well as levels of stress hormones like cortisol, disrupting normal sleep patterns. Moreover, reduced activity of the parasympathetic nervous system diminishes the ability to achieve a state of physical relaxation and recovery at night, further exacerbating sleep disorders.^[26]^

Rybel W-R’s comparison of ANS function between chronic insomnia patients and healthy controls aimed to explore the possibility of diagnosing insomnia through physiological parameters monitored by wearable devices.^[27]^ Findings revealed that insomnia patients have significantly lower sleep efficiency and total sleep time compared to healthy controls, with longer sleep latency and increased time awake after sleep onset. Notably, insomnia patients exhibit significant differences in deep sleep activity, with no significant differences during REM sleep stages. Additionally, insomnia patients generally have higher heart rates across all sleep stages compared to controls, with decreased heart rate variability during deep sleep and increased during REM sleep, and also a significant increase in sweating. These findings uncover a pronounced enhancement of autonomic nervous system activity during sleep in insomnia patients, manifested by increased heart rate, abnormal temperature regulation, and increased sweating.

Similarly, under pathological conditions, dysfunction in specific areas of the central nervous system (CNS), especially in the cerebral cortex and particularly in the frontal lobes, is closely associated with the development of insomnia.^[28]^ This persistent state of heightened arousal may be caused by daytime psychological stress or chronic physiological changes due to prolonged stress, disrupting the balance of neurotransmitters and hormones affecting sleep.^[29]^ A study^[30]^ aimed to explore the relationship between the CNS’s excitability and insomnia, and how emotional dimensions mediate this relationship. The study found a negative correlation between excitability and insomnia symptoms, and a positive correlation with pleasant emotions and energy arousal, and a negative correlation with tension arousal. These findings emphasize the importance of assessing and understanding the role of CNS properties in the onset of insomnia, particularly the interaction between excitability and arousal-related emotional dimensions.

Dysfunctions in the hypothalamus and pineal gland, such as imbalances in melatonin secretion and sleep-wake cycle regulation, are also common pathological features of insomnia.^[31]^ Factors like light pollution and circadian rhythm disruption may further interfere with these areas’ functions.^[32]^ The hypothalamus plays a central role in sleep-wake regulation through its communication with different brain regions. Ding S’s study^[33]^ linked neuroimaging findings with PSQI scores to investigate how functional connectivity disturbances in the hypothalamus participate in the pathological process of insomnia disorders. Compared to healthy controls, insomnia patients showed significantly increased resting-state functional connectivity between the left hypothalamus and several brain regions, including the medial prefrontal cortex, while connectivity with the right temporal cortex decreased. This resting-state functional connectivity intensity positively correlated with PSQI scores, revealing enhanced functional connectivity between the left hypothalamus and several brain regions, including the medial prefrontal cortex, in insomnia patients.

### 3.3. Insufficient blood perfusion

Insufficient blood perfusion manifests as a reduction in local or systemic blood flow and is identified as a potential pathophysiological mechanism affecting sleep. The reduced blood flow leads to insufficient oxygen and nutrient supply to the brain, which affects the balance of neurotransmitters and brain functions crucial for controlling the sleep cycle, including the regulation and maintenance of sleep.^[34]^ Inadequate blood supply can also cause nocturnal discomfort or pain, such as leg pain, and symptoms related to cardiovascular diseases like chest pain or difficulty breathing, which may lead to frequent awakenings at night, further disrupting sleep.^[35]^ Moreover, good blood circulation is vital for entering and maintaining deep sleep stages. Therefore, insufficient perfusion may reduce sleep quality, shorten the duration of deep sleep, and impact the body’s recovery processes and functional restoration. This series of mechanisms reveals the complex relationship between insufficient blood perfusion and insomnia, highlighting the importance of maintaining proper blood circulation for a healthy sleep pattern.^[36]^

In a study,^[37]^ researchers explored the mechanisms behind the co-occurrence of chronic insomnia disorder and major depressive disorder. Participants’ levels of insomnia and depression were assessed through questionnaires, and their brain blood perfusion and functional connectivity values were measured using brain imaging techniques to analyze the correlation of these values with the severity of insomnia and depression. The study found that compared to the healthy control group, the patient group had reduced blood flow in the cerebellum, vermis, right hippocampus, and left parahippocampal gyrus, negatively correlating with the severity of insomnia and depression. At the same time, the connectivity between the left cerebellum-right caudate and right hippocampus-left inferior frontal gyrus increased, positively correlating with the severity of insomnia and depression. On the other hand, the connectivity between the left cerebellum-left fusiform gyrus, left cerebellum-left occipital lobe, right hippocampus-right paracentral lobule, and right hippocampus-right precentral gyrus decreased, also related to insomnia or depression. The results suggest that changes in brain blood flow and functional connectivity in patients with insomnia and depression, especially in the cerebellum and hippocampal regions, reflect abnormalities in sleep and emotional regulation.

Yan W’s study^[38]^ aimed to reveal the interrelation among cerebral blood flow perfusion insufficiency, differences in sleep quality, and insomnia in patients with cerebral small vessel disease. Particularly, arterial spin labeling technology revealed a significant reduction in gray matter perfusion in the left hippocampal region in patients with poor sleep quality, negatively correlated with the PSQI scores. This finding emphasizes the direct association between insufficient cerebral blood flow perfusion, poor sleep quality, and insomnia, providing a new perspective for understanding the interplay between sleep disorders and cerebral vascular health.

## 4. Acupuncture

Acupuncture, an ancient and unique non-pharmacological treatment method, demonstrates significant therapeutic effects on patients with insomnia by inserting and rotating needles at specific acupoints to stimulate the human nervous system.^[39]^ This treatment primarily regulates neurotransmitters and neurohormones to improve sleep quality and alleviate stress.^[40]^ It also relaxes vascular smooth muscles and dilates blood vessels to enhance blood circulation, promoting overall relaxation and thereby aiding in the onset of sleep. Furthermore, acupuncture can adjust the endocrine system, for instance, by modulating the pituitary-adrenal axis to affect cortisol levels, thereby reducing stress-induced insomnia.^[41]^ In addition, it promotes muscle relaxation,^[42]^ regulates the immune system,^[43]^ and improves overall health and psychological well-being, offering a comprehensive treatment approach for patients. Although the precise mechanisms of acupuncture in treating insomnia are still under investigation, clinical practices and studies have proven its effectiveness in improving symptoms of insomnia through multiple pathways, bringing psychological and physiological benefits to patients.

### 4.1. Regulating neurotransmitters

Acupuncture can activate the central nervous system, increasing the availability of tryptophan and the level of 5-HT in the blood. As a precursor to the synthesis of 5-HT, the increase of tryptophan in the blood promotes the synthesis and release of 5-HT in the brain.^[44]^ 5-HT, a key inhibitory neurotransmitter, is involved in regulating the sleep cycle, mood, and pain perception.^[45]^ By enhancing the level of 5-HT, acupuncture helps alleviate symptoms of anxiety and depression, thereby improving insomnia.^[46]^ Acupuncture can also promote sleep by lowering the levels of cortisol in the stress response. Elevated levels of cortisol can inhibit the activity of 5-HT, further worsening sleep quality.^[47]^ Acupuncture reduces the body’s stress response by regulating the activity of the hypothalamic-pituitary-adrenal axis and decreasing the release of cortisol, aiding in the restoration of normal sleep patterns.^[48]^ Thus, by regulating the levels of 5-HT and cortisol, acupuncture improves sleep structure, extends the deep sleep phase, reduces the number of awakenings at night, and enhances sleep efficiency, which is crucial for improving the overall sleep quality of patients with insomnia.

In a study^[40]^ exploring the effectiveness of acupuncture in treating chronic insomnia and accompanying symptoms of anxiety and depression, patients receiving acupuncture treatment showed more significant reductions in serum cortisol levels and increases in 5-HT compared to those receiving placebo treatment. This finding reveals the potential link between abnormalities in serum cortisol and 5-HT levels and emotional regulation disorders in patients with chronic insomnia. Further analysis demonstrated that acupuncture significantly improved patients’ sleep quality, efficiency, and onset time, and effectively relieved symptoms of anxiety and depression. Comparing the treatment effects of the 2 groups, the real acupuncture treatment group not only showed more significant improvements but also longer-lasting effects, indicating that acupuncture has significant long-term efficacy in treating chronic insomnia and its comorbid emotional disorders compared to placebo treatment.

Another study^[49]^ aimed to observe the clinical efficacy of “Tongdu Tiaoshen” acupuncture treatment for chronic insomnia and its impact on patients’ serum GABA and cortisol levels, exploring the potential mechanisms by which acupuncture improves insomnia. By randomly dividing 60 patients with chronic insomnia into an observation group receiving acupuncture treatment and a control group taking estazolam tablets, the study detailed the implementation of the two treatment methods, including the selection of acupuncture points and treatment frequency, as well as the dosage and administration of estazolam, over a 4-week treatment period. The results showed significant improvements in the PSQI scores, serum GABA, and cortisol levels in both groups after treatment, with the clinical efficacy of acupuncture treatment comparable to that of estazolam. Notably, acupuncture treatment significantly outperformed estazolam treatment in improving daytime functional impairment.

### 4.2. Regulating the nervous system

Li’s research^[50]^ reviewed the foundational and clinical studies on the effects of acupuncture on the regulation of the ANS and the maintenance of internal balance. The research found that the ANS is one of the key neural pathways in the signal transmission of acupuncture, which can alleviate diseases and symptoms accompanied by autonomic nervous dysfunction, such as insomnia, migraines, depression, functional dyspepsia, and constipation.

Akita et al^[51]^ explored the impact of acupuncture on autonomic nervous system indicators – HRV during sleep in healthy adult males. The study compared the effects of real and sham acupuncture stimulation on HRV indicators through a crossover design. Researchers estimated sleep stages by combining HRV markers (HRV Sleep Index [Hsi]) during NREM sleep and physical activity recorded by a motion monitor. The results showed that, compared to sham acupuncture, real acupuncture significantly lowered the heart rate throughout the sleep period and significantly increased the power of low and high frequency components of HRV. Especially during the NREM sleep phase, real acupuncture compared to sham acupuncture, also showed a reduction in heart rate and an increase in HRV component power; however, during REM sleep, although the heart rate was lower after real acupuncture, there was no significant difference in HRV indicators. This suggests that acupuncture may particularly enhance parasympathetic nervous activity during NREM sleep, providing objective physiological evidence that acupuncture improves sleep quality, especially by increasing parasympathetic HRV indicators during NREM sleep.

Cao et al^[52]^ studied the effects of electroacupuncture on a rat model of insomnia, observing the behavioral and physiological changes in rats with insomnia, aiming to explore how acupuncture affects sleep by altering various biological pathways essential for the function of the central nervous system. The study induced an insomnia model in rats by administering p-chlorophenylalanine (PCPA) and then treated specific acupoints with electroacupuncture to evaluate its effects on rat behavior, neurotransmitter levels, histopathology, and expression of sleep-related factors. The experimental results revealed that electroacupuncture could reduce apoptosis in neurons in the hippocampus and brainstem and alleviate insomnia by regulating the PI3K/Akt and cAMP/CREB/BDNF pathways. This effect involved not only the adjustment of neurotransmitter levels, such as reducing the release of excitatory monoamine neurotransmitters, but also involved broader signaling pathways, effectively lowering excitability in the rat central nervous system and improving sleep quality. Acupuncture helps alleviate symptoms of insomnia by reducing the release of excitatory neurotransmitters like 5-HT, dopamine, Norepinephrine. Moreover, acupuncture reduces neuronal apoptosis by increasing BCL-2 expression and decreasing the expression of BAX, BAD, and Caspase-3. By activating the PI3K/AKT and CAMP/CREB pathways and increasing BDNF expression, acupuncture further promotes the survival and function of neurons. Notably, acupuncture can also regulate sleep-related immune function indicators, including raising the levels of IL-1β, PGD2, melatonin, while lowering IL-6, TNF-α, IFN-γ, and cortisol levels, improving symptoms of insomnia by modulating immune responses and reducing inflammatory reactions.

### 4.3. Anti-inflammatory

Another mechanism of acupuncture in the treatment of insomnia is its anti-inflammatory effect. Inflammation is considered a potential mechanism of insomnia, especially since a chronic inflammatory state is associated with various sleep disorders.^[53]^ When exploring the mechanisms of acupuncture treatment for insomnia, it is particularly noteworthy for its role in regulating the body’s inflammatory response. Acupuncture can effectively reduce the levels of inflammatory cytokines in the body, including TNF-α and interleukins. These inflammatory cytokines play a crucial role in regulating the body’s response to inflammation and in the formation of chronic inflammatory states.^[54]^ On the other hand, acupuncture can also promote the release of anti-inflammatory factors in the body. Anti-inflammatory factors can counteract the effects of inflammatory factors, and by increasing the levels of anti-inflammatory factors, acupuncture helps to control the body’s inflammatory response, thereby playing a role in combating insomnia.^[55]^

Researchers^[56]^ prepared an insomnia rat model by intraperitoneal injection of PCPA in rats, followed by acupuncture treatment, aiming to explore how acupuncture improves symptoms of insomnia by reducing inflammation and inhibiting the ERK/NF-κB signaling pathway. Additionally, the study investigated changes in mRNA and protein expression related to the ERK/NF-κB signaling pathway in the hippocampus. The results showed that acupuncture treatment not only significantly prolonged the sleep duration of the insomnia model rats but also improved their general state and behavioral responses, and enhanced learning ability and spatial memory. More importantly, acupuncture treatment significantly reduced the levels of inflammatory factors in rat serum and hippocampus. These inflammatory factors play a key role in regulating the sleep-wake cycle and the interaction with the immune system, indicating that acupuncture may improve sleep quality by regulating these key inflammatory factors. One of the key findings of the study is that acupuncture could inhibit the expression of mRNA and proteins of key components of the ERK/NF-κB signaling pathway in the rat hippocampus. This result provides important clues to the potential molecular mechanisms by which acupuncture relieves symptoms of insomnia, revealing that acupuncture may exert its anti-inflammatory therapeutic effects by inhibiting the ERK/NF-κB signaling pathway. In another randomized controlled trial,^[57]^ researchers also considered inflammation parameters as one of the diagnostic and treatment outcomes for patients with insomnia. The study found that compared to the psychoeducation group, the acupuncture group showed improvements in sleep quality, stress, anxiety, and fatigue.

## 5. Conclusions

Acupuncture treatment for insomnia is a time-honored and effective method, involving multiple biological pathways. By regulating neurotransmitter levels, improving neuroendocrine balance, promoting neuroplasticity, and exerting anti-inflammatory actions, acupuncture plays a crucial role in improving sleep quality. Overall, acupuncture treatment offers a safe and effective alternative or adjunctive therapy, bringing new hope to patients with insomnia. Although it may benefit some individuals, the effectiveness of this method varies from person to person and comes with certain limitations and potential risks. Firstly, the quality and quantity of research on acupuncture treatment are still insufficient for it to be recognized as a widely accepted medical treatment. Secondly, improper practices may lead to side effects such as infection, bleeding, and needle injuries. Moreover, acupuncture treatment typically requires multiple sessions, and each session might be lengthy, which could inconvenience patients. Finally, since acupuncture treatment is deeply influenced by Traditional Chinese Medicine philosophy, it may not align with the cultural background and belief systems of some individuals, potentially affecting their acceptance and treatment outcomes. Therefore, when considering acupuncture treatment, it is advisable to consult a healthcare professional to ensure the therapy is suitable for one’s health condition.

## Author contributions

**Conceptualization:** Haishen Zhao.

**Methodology:** Chengjun Liu.

**Supervision:** Hong Ye.

**Validation:** Kejun Shi.

**Writing – original draft:** Yijie Yao.

**Writing – review & editing:** Manya Wang.

## References

[1] Perlis ML Posner D Riemann D Bastien CH Teel J Thase M. Insomnia. Lancet. 2022;400:1047–60. 36115372 10.1016/S0140-6736(22)00879-0

[2] Nyhuis C Fernandez-Mendoza J. Insomnia nosology: a systematic review and critical appraisal of historical diagnostic categories and current phenotypes. J Sleep Res. 2023;32:e13910. 37122153 10.1111/jsr.13910 PMC11948287

[3] Górska K Ścisło L Pluta J Antoniak-Sobczak K Fojcik J Górski MK. What’s new in sleep disorders? Characteristics of sleep disorders according to ICD-11. Nurs Problems/Problemy Pielęgniarstwa. 2023;31:159–65.

[4] Sutton EL. Insomnia. Ann Intern Med. 2021;174:ITC33–48. 33683929 10.7326/AITC202103160

[5] Wang Z Huang Y Wang S. A case report of fatal familial insomnia with cerebrospinal fluid leukocytosis during the COVID-19 epidemic and review of the literature. Prion. 2024;1:1–10. 10.1080/19336896.2023.2298520 PMC11654706 38226945

[6] Savin KL Clark TL Perez-Ramirez P Allen TS Tristão Parra M Gallo LC. The effect of cognitive behavioral therapy for insomnia (CBT-I) on Cardiometabolic health biomarkers: a systematic review of randomized controlled trials. Behav Sleep Med. 2023;21:671–94. 36476211 10.1080/15402002.2022.2154213 PMC10244489

[7] Riemann D Espie CA Altena E. The European insomnia guideline: an update on the diagnosis and treatment of insomnia 2023. J Sleep Res. 2023;32:e14035. 38016484 10.1111/jsr.14035

[8] Morin CM Jarrin DC. Epidemiology of insomnia: prevalence, course, risk factors, and public health burden. Sleep Med Clinics. 2022;17:173–91. 10.1016/j.jsmc.2022.03.003 35659072

[9] Zeng L-N Zong Q-Q Yang Y. Gender difference in the prevalence of insomnia: a meta-analysis of observational studies. Front Psychiatry. 2020;11:577429. 33329116 10.3389/fpsyt.2020.577429 PMC7714764

[10] Benussi A Premi E Gazzina S. Neurotransmitter imbalance dysregulates brain dynamic fluidity in frontotemporal degeneration. Neurobiol Aging. 2020;94:176–84. 32629312 10.1016/j.neurobiolaging.2020.05.017

[11] Zhang R Yang Y-s Liu X-c. Correlation study of basic Chinese medicine syndromes and neurotransmitter levels in patients with primary insomnia. Chin J Integr Med. 2016;1:5. 10.1007/s11655-016-2752-2 28028724

[12] Di Palma M Catalano M Serpe C. Lipopolysaccharide augments microglial GABA uptake by increasing GABA transporter‐1 trafficking and bestrophin‐1 expression. Glia. 2023;71:2527–40. 37431178 10.1002/glia.24437

[13] Favuzzi E Huang S Saldi GA. GABA-receptive microglia selectively sculpt developing inhibitory circuits. Cell. 2021;184:4048–63.e32. 34233165 10.1016/j.cell.2021.06.018 PMC9122259

[14] Vargas-Parada A Loeza-Alcocer E González-Ramírez R. γ-Aminobutyric acid (GABA) from satellite glial cells tonically depresses the excitability of primary afferent fibers. Neurosci Res. 2021;170:50–8. 32987088 10.1016/j.neures.2020.08.007

[15] Kim S Jo K Hong K-B Han SH Suh HJ. GABA and l-theanine mixture decreases sleep latency and improves NREM sleep. Pharm Biol. 2019;57:64–72. 10.1080/13880209.2018.1557698 PMC6366437 30707852

[16] Xiang T Liao J Cai Y. Impairment of GABA inhibition in insomnia disorders: evidence from the peripheral blood system. Front Psychiatry. 2023;14:1134434. 36846238 10.3389/fpsyt.2023.1134434 PMC9947704

[17] Zhang X-H Zhang X Liu X-W. Examining the Role of GLU/GABA to GLN metabolic cycle in the pathogenesis of post-stroke depressive disorder and insomnia. Neuropsychiatr Dis Treat. 2023;19:2833–40. 38149001 10.2147/NDT.S443844 PMC10750479

[18] Bolattürk OF İsmailoğullari S Aldemir R Tokmakçi M Ulusoy EK. The correlation between the electroencephalographic spectral analysis and salivary cortisol rhythm in insomnia. Sleep Breath. 2020;24:661–7. 32062753 10.1007/s11325-020-02032-1

[19] Passos GS Youngstedt SD Rozales AARC. Insomnia severity is associated with morning cortisol and psychological health. Sleep Sci. 2023;16:092–6. 10.1055/s-0043-1767754 PMC10157827 37151768

[20] Roehrs T Roth T. Hyperarousal in insomnia: pre-sleep and diurnal cortisol levels in response to chronic zolpidem treatment. Sleep Med. 2019;61:52–6. 31255482 10.1016/j.sleep.2019.04.010 PMC6721980

[21] Philippe DD Giuseppe DG. Serotonin in Health and Disease. Int J Mol Sci. 2020;21. 10.3390/ijms21103500 PMC7278959 32429111

[22] Mohammad-Ali S Hamed F Mohadeseh T. Parkinson’s disease: a narrative review on potential molecular mechanisms of sleep disturbances, REM behavior disorder, and melatonin. Brain Sci. 2023;13. 10.3390/brainsci13060914 PMC10296587 37371392

[23] Cristian F-P Ștefania D Diana T Oana F-P. Neurobiology of sleep (Review). Exp Ther Med. 2021;21. 10.3892/etm.2021.9703 PMC7851648 33603879

[24] Eleonora T Giorgio C Monica S. Sleep, sleep deprivation, autonomic nervous system and cardiovascular diseases. Neurosci Biobehav Rev. 2016;74. 10.1016/j.neubiorev.2016.07.004 27397854

[25] Yue C Zhaoyang H Min C. Dysfunction of the cardiac parasympathetic system in fatal familial insomnia: a heart rate variability study. Sleep. 2022;46. 10.1093/sleep/zsac294 PMC10091088 36472576

[26] Eduardo EB. Physiology and Pathophysiology of the autonomic nervous system. Continuum (Minneap Minn). 2020;26. 10.1212/CON.0000000000000817 31996619

[27] Rybel W-R Javier G-G Marta V-A. Monitoring differences in the function of the autonomic nervous system in patients with chronic insomnia using a wearable device. Sleep Med. 2024;115. 10.1016/j.sleep.2024.02.017 38359591

[28] Katrina LG Neda J Jodie NP. The genetic architecture of the human cerebral cortex. Science. 2020;367. 10.1126/science.aay6690 PMC7295264 32193296

[29] Marcio Luciano de Souza B Raimundo Nonato Delgado R Ricardo Oliveira S. The hypothalamic-pituitary-adrenal axis and the central monoaminergic systems: a pathophysiological link to insomnia with clinical implications. Sleep Sci. 2022;15. 10.5935/1984-0063.20220032 PMC9153981 35662967

[30] Włodzimierz O Magdalena O. Strength of excitation and insomnia as mediated by mood dimensions. Curr Issues Personal Psychol. 2022;11. 10.5114/cipp/151671 PMC10654329 38013831

[31] Sebastiaan O Van Litsenburg RRL Paul JR. Sleep disorders and the hypothalamus. Handb Clin Neurol. 2021;182. 10.1016/B978-0-12-819973-2.00025-3 34266606

[32] Nicola Luigi B Sergio G Luca P. Planetary sleep medicine: studying sleep at the individual, population, and planetary level. Front Public Health. 2022;10. 10.3389/fpubh.2022.1005100 PMC9624384 36330122

[33] Shuang D Lijuan G Hanjiaerbieke K. Novel neuroimaging biomarker for sleep quality in insomnia disorder: a hypothalamus resting state study. Front Neurosci. 2021;15. 10.3389/fnins.2021.634984 PMC7953135 33716655

[34] Jooyeon JI Hyeonseok SJ Jong-Sik P. Associations between brain perfusion and sleep disturbance in patients with Alzheimer’s Disease. Dement Neurocogn Disord. 2017;16. 10.12779/dnd.2017.16.3.72 PMC6427985 30906374

[35] Christian G Jr Jeremy BR Michael F Jason C Susan RW. ECMO and right ventricular failure: review of the literature. J Intensive Care Med. 2020;36. 10.1177/0885066619900503 31964208

[36] Galina Y Olga B Andrey T. The glymphatic system and meningeal lymphatics of the brain: new understanding of brain clearance. Rev Neurosci. 2021;32. 10.1515/revneuro-2020-0106 33618444

[37] Minghe X Qian W Bo L. Cerebellum and hippocampus abnormalities in patients with insomnia comorbid depression: a study on cerebral blood perfusion and functional connectivity. Front Neurosci. 2023;17. 10.3389/fnins.2023.1202514 PMC10311636 37397441

[38] Wei Y Duanlu H Zhixin L Weijun T Xiang H Yuping T. Reduced left hippocampal perfusion is associated with insomnia in patients with cerebral small vessel disease. CNS Spectr. 2023;28. 10.1017/S1092852923002250 37095715

[39] Melissa LH Marita GT Laura MS. Acupuncture and acupressure for dementia behavioral and psychological symptoms: a scoping review. West J Nurs Res. 2019;42. 10.1177/0193945919890552 PMC7272277 31802723

[40] Liu C Zhao Y Qin S Wang X Jiang Y Wu W. Randomized controlled trial of acupuncture for anxiety and depression in patients with chronic insomnia. Ann Transl Med. 2021;9. 10.21037/atm-21-3845 PMC8506741 34733978

[41] Jing-Qi F Wei-Jing L Wei-Qiang T. Effectiveness of acupuncture for anxiety among patients with parkinson disease: a randomized clinical trial. JAMA Netw Open. 2022;5. 10.1001/jamanetworkopen.2022.32133 PMC9494193 36129711

[42] George GAP Michael M Akhil M Davong David P Neil PS Adam IP. Acupuncture in Sports Medicine. J Acupunct Meridian Stud. 2023;16. 10.51507/j.jams.2023.16.6.239 38115589

[43] Meng W Weili L Jiayi G Shenbin L. The immunomodulatory mechanisms for acupuncture practice. Front Immunol. 2023;14. 10.3389/fimmu.2023.1147718 PMC10117649 37090714

[44] Bao C Zhong J Liu H. [Effect of acupuncture-moxibustion on negative emotions and plasma tryptophan metabolism in patients with Crohn’s disease at active stage]. Zhongguo Zhen Jiu. 2021;41:17–22. 33559436 10.13703/j.0255-2930.20200814-k0003

[45] Feng X Huang K Chen L Zhou K. Clinical efficacy of the shallow puncture and more-twirling acupuncture method in migraine treatment and its effects on serum 5-HT and β-EP levels. Technol Health Care. 2023;31:533–40. 37038799 10.3233/THC-236047 PMC10200134

[46] Yan L Li Z Zhang Y. Shugan Tiaoshen[Effect of acupuncture combined with western medication on depression-insomnia comorbidity due to COVID-19 quarantine: a multi-central randomized controlled trial]. Zhongguo Zhen Jiu. 2023;43:255–60. 36858384 10.13703/j.0255-2930.20221030-k0004

[47] Houser M Coborn J Sinton C Perez-Leighton C Teske J. Sleep loss in male rats contributes more to weight gain during sleep disruption than stress assessed by corticosterone. Neurosci Lett. 2023;792:136959. 36370954 10.1016/j.neulet.2022.136959 PMC9710233

[48] Yu X Yang W Zhao N. [Acupuncture for delayed sleep-wake phase disorder: a randomized controlled trial]. Zhongguo Zhen Jiu. 2023;43:245–51. 36858383 10.13703/j.0255-2930.20221031-k0002

[49] Wu W Zheng S Liu C. Tongdu Tiaoshen [Effect of acupuncture on serum GABA and CORT levels in patients with chronic insomnia]. Zhongguo Zhen Jiu. 2021;41:721–4. 34259401 10.13703/j.0255-2930.20200704-k0001

[50] Li Y Li W Wang S. The autonomic nervous system: a potential link to the efficacy of acupuncture. Front Neurosci. 2022;16:1038945. 36570846 10.3389/fnins.2022.1038945 PMC9772996

[51] Akita T Kurono Y Yamada A Hayano J Minagawa M. Effects of acupuncture on autonomic nervous functions during sleep: comparison with nonacupuncture site stimulation using a crossover design. J Integr Complement Med. 2022;28:791–8. 35895512 10.1089/jicm.2022.0526

[52] Cao F Xu Y Zhang M. Baihui (DU20), Shenmen (HT7) and Sanyinjiao (SP6) target the cAMP/CREB/BDNF and PI3K/Akt pathways to reduce central nervous system apoptosis in rats with insomnia. Heliyon. 2022;8:e12574. 36636219 10.1016/j.heliyon.2022.e12574 PMC9830165

[53] Zhai S Li T Zhang D. Insomnia trajectories predict chronic inflammation over 2 years at the transition to adulthood. J Sleep Res. 2023;32:e13906. 37062708 10.1111/jsr.13906

[54] Yang S Cho S Kwon S. Acupuncture attenuates postoperative inflammation in patients after craniotomy: a prospective, open-label, controlled trial. Medicine (Baltimore). 2020;99:e19071. 32176032 10.1097/MD.0000000000019071 PMC7440145

[55] Wang Z Yi T Long M. Electro-Acupuncture at Zusanli Acupoint (ST36) suppresses inflammation in allergic contact dermatitis via triggering Local IL-10 production and inhibiting p38 MAPK Activation. Inflammation. 2017;40:1351–64. 28493082 10.1007/s10753-017-0578-5

[56] Zhang M Zhao J Li Z Shao J Gao X. Acupuncture at Back-Shu point improves insomnia by reducing inflammation and inhibiting the ERK/NF-κB signaling pathway. World J Psychiatry. 2023;13:340–50. 37383281 10.5498/wjp.v13.i6.340 PMC10294136

[57] Höxtermann M Buner K Haller H. Efficacy and safety of auricular acupuncture for the treatment of insomnia in breast cancer survivors: a randomized controlled trial. Cancers. 2021;13. 10.3390/cancers13164082 PMC8394534 34439234

